# Lessons Learned and Future Directions of MetaTutor: Leveraging Multichannel Data to Scaffold Self-Regulated Learning With an Intelligent Tutoring System

**DOI:** 10.3389/fpsyg.2022.813632

**Published:** 2022-06-14

**Authors:** Roger Azevedo, François Bouchet, Melissa Duffy, Jason Harley, Michelle Taub, Gregory Trevors, Elizabeth Cloude, Daryn Dever, Megan Wiedbusch, Franz Wortha, Rebeca Cerezo

**Affiliations:** ^1^School of Modeling Simulation and Training, University of Central Florida, Orlando, FL, United States; ^2^Laboratoire d’Informatique de Paris 6 (LIP6), Sorbonne Université, Paris, France; ^3^Educational Studies, University of South Carolina, Columbia, SC, United States; ^4^Faculty of Medicine, McGill University, Montreal, QC, Canada; ^5^Research Institute of the McGill University Health Center, Montreal, QC, Canada; ^6^Soar Technology, Inc., Orlando, FL, United States; ^7^Institute of Psychology, University of Greifswald, Greifswald, Germany; ^8^Department of Psychology, University of Oviedo, Oviedo, Spain

**Keywords:** self-regulated learning, learning, multimodal data, intelligent tutoring systems, scaffolding, metacognition, trace data, pedagogical agents

## Abstract

Self-regulated learning (SRL) is critical for learning across tasks, domains, and contexts. Despite its importance, research shows that not all learners are equally skilled at accurately and dynamically monitoring and regulating their self-regulatory processes. Therefore, learning technologies, such as intelligent tutoring systems (ITSs), have been designed to measure and foster SRL. This paper presents an overview of over 10 years of research on SRL with MetaTutor, a hypermedia-based ITS designed to scaffold college students’ SRL while they learn about the human circulatory system. MetaTutor’s architecture and instructional features are designed based on models of SRL, empirical evidence on human and computerized tutoring principles of multimedia learning, Artificial Intelligence (AI) in educational systems for metacognition and SRL, and research on SRL from our team and that of other researchers. We present MetaTutor followed by a synthesis of key research findings on the effectiveness of various versions of the system (e.g., adaptive scaffolding vs. no scaffolding of self-regulatory behavior) on learning outcomes. First, we focus on findings from self-reports, learning outcomes, and multimodal data (e.g., log files, eye tracking, facial expressions of emotion, screen recordings) and their contributions to our understanding of SRL with an ITS. Second, we elaborate on the role of embedded pedagogical agents (PAs) as external regulators designed to scaffold learners’ cognitive and metacognitive SRL strategy use. Third, we highlight and elaborate on the contributions of multimodal data in measuring and understanding the role of cognitive, affective, metacognitive, and motivational (CAMM) processes. Additionally, we unpack some of the challenges these data pose for designing real-time instructional interventions that scaffold SRL. Fourth, we present existing theoretical, methodological, and analytical challenges and briefly discuss lessons learned and open challenges.

## Introduction: Self-Regulated Learning and Advanced Learning Technologies

Self-regulated learning (SRL) is essential to learning, reasoning, and problem-solving across tasks, domains, and contexts (Pintrich, 2000; Zimmerman and Schunk, 2011; Panedero, 2017; Schunk and Greene, 2018). However, research shows that learners experience challenges in accurately, dynamically, and effectively monitoring and regulating their cognitive, affective, metacognitive, motivational, and social self-regulatory processes. A solution to this challenge has been designing and implementing learning technologies such as intelligent tutoring systems (ITSs) to measure and foster SRL (Azevedo and Aleven, 2013). This paper presents an overview of over 10 years of research on SRL with MetaTutor, a hypermedia-based ITS designed to scaffold college students’ SRL while they learn about the human circulatory system. MetaTutor’s architecture and instructional features are designed based on Winne (2018; 2020) model of SRL, empirical evidence on human (Azevedo et al., 2008; Chi, 2021) and computerized tutoring (Nye et al., 2014; du Boulay and Luckin, 2016; Johnson and Lester, 2016, 2018; Graesser, 2020), AI in educational systems for metacognition and SRL (Aleven and Koedinger, 2002; Azevedo and Aleven, 2013; Biswas et al., 2016; Azevedo and Wiedbusch, in press), Mayer and Fiorella (in press) principles of multimedia learning, and extensive research on SRL, ITSs, serious games, simulations, and open-ended hypermedia from our team and other researchers (Bannert et al., 2014; Biswas et al., 2018; Schunk and Greene, 2018; Azevedo et al., 2019; Sonnenberg and Bannert, 2019; Lajoie, 2021).

We present a synthesis of key research findings and the effectiveness of different versions of the system (e.g., adaptive scaffolding vs. no scaffolding of self-regulatory behavior) on learning outcomes. First, we focus on findings from self-reports, learning outcomes, and multimodal data (e.g., log files, eye tracking, facial expressions of emotion, and screen recordings) and their contributions to our understanding of SRL with an ITS. Second, we elaborate on the role of embedded PAs as external regulators designed to scaffold learners’ cognitive and metacognitive SRL strategies. Third, we highlight and discuss the contributions of multimodal data in measuring and understanding the role of cognitive, affective, metacognitive, and motivational (CAMM) processes while unpacking the challenges these data pose for designing real-time instructional interventions that scaffold SRL. Fourth, we present existing theoretical, methodological, and analytical challenges and briefly discuss lessons learned with MetaTutor and open challenges.

We briefly describe Winne and Hadwin’s model of SRL to contextualize our program of research investigating SRL and MetaTutor with college students over dozens of studies. We utilized Winne and Hadwin’s information processing theory (IPT) of SRL (Winne and Hadwin, 1998, 2008; Winne, 2018) extensively in our research on MetaTutor. The theory states that learning occurs through a series of four cyclical phases, where metacognitive monitoring and control are the hubs of SRL. These processes are captured as events that unfold over time and across several phases. This model is appropriate because we view metacognition as a series of events (e.g., planning → cognitive strategy A → metacognitive monitoring process C → cognitive strategy F → metacognitive monitoring process W →…) that occur during learning. Specifically, the four phases involve (1) understanding the task, (2) setting goals and making plans to accomplish goals, (3) applying learning strategies for making progress based on (2), and (4) adapting to (2–3) as new challenges and demands emerge. In the context of MetaTutor studies, these phases would include understanding the overall learning goal provided by the system (e.g., *“you have 45 minutes to learn all you can about the human circulatory system. Make sure you learn about all the components, how they work together, and how they help support the healthy functioning of the human body”*). Once the learner understands the task, they would then be expected to generate several learning subgoals (e.g., learn how the pulmonary and systemic systems work in tandem to support the human body) to accomplish the overall learning objectives. Learners could track and accomplish their subgoals and learning objectives using MetaTutor’s interface features (e.g., SRL palette to indicate to the system which SRL processes they were planning on using) and with the support of the four PAs. Self-regulating in the context of learning with MetaTutor meant learners had to use cognitive and metacognitive processes to accomplish their sub-goals such as making inferences, summarizing, making hypotheses, and others while metacognitively monitoring their learning by engaging in judgments of learning (JOL), feelings-of-knowing (FOK), monitoring progress toward goals, and evaluating the relevance of content, such text and diagrams, given their current learning goal. While self-regulating with MetaTutor, we expected learners to also experience emotional and motivational states that were captured by multimodal data using cameras, physiological devices, and embedded self-report measures.

The effectiveness of the system has been extensively tested and published widely in several cognitive, learning, instructional, and computer science refereed conference proceedings, journals, chapters, and widely disseminated at national and international conferences (Azevedo et al., 2010, Azevedo et al., 2013, 2018, 2019). MetaTutor was originally designed to be both a learning tool to foster self-regulation and a research tool to collect trace data on CAMM processes as they unfolded during learning. The system supports several learning strategies through its user interface including features that prompt learners to activate prior knowledge about content, goal setting, evaluating learning strategies, integrating information across diagrams, evaluating content, summarizing key information, note-taking, and drawing. It also scaffolds specific metacognitive monitoring processes, such as JOLs and FOKs. The unique contribution of this paper is its comprehensiveness and synthesis of all the studies conducted by our team and collaborators over more than a decade that emphasizes empirical findings across CAMM processes.

The central research questions addressed in our research on MetaTutor with predominantly Caucasian female college students, include: (1) How do different scaffolding methods influence students’ learning about human biology and their SRL performance? (2) How do different scaffolds influence students’ deployment, effectiveness, and quality of SRL processes during learning with MetaTutor? (3) What is the temporal and dynamic nature of students’ CAMM processes while using MetaTutor to learn about complex biology topics with MetaTutor? (4) Do process-oriented multimodal trace data (e.g., log files, concurrent verbalizations, eye movements, facial expressions of emotion, and physiological sensors) reveal “signatures” of specific cognitive and metacognitive processes [e.g., ease-of-learning (EOL), JOLs]? and (5) To what extent do self-report and process-oriented multimodal trace data predict SRL behaviors, learning, and performance, based on experimental conditions and individual differences?

## MetaTutor: A Hypermedia-Based Intelligent Tutoring System for Human Biology

**FIGURE 1:**
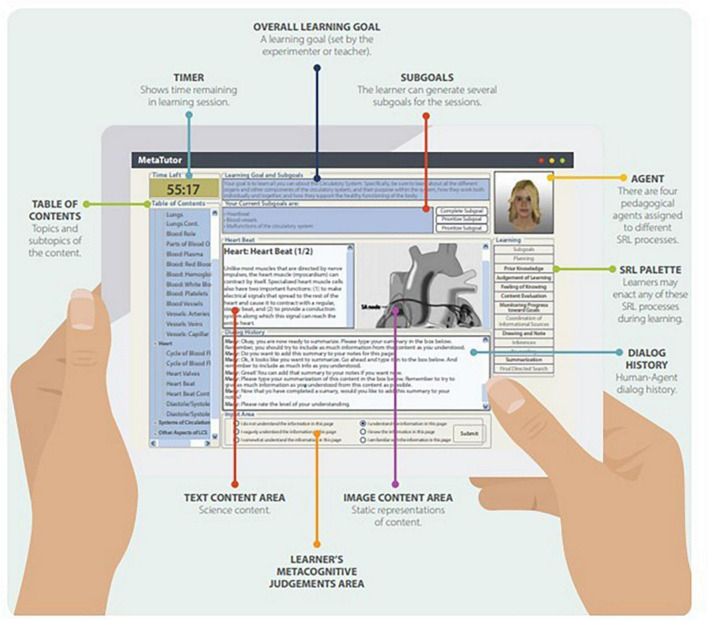
MetaTutor’s main interface elements.

MetaTutor is a hypermedia-based ITS that teaches challenging STEM content (e.g., human circulatory system) developed by Azevedo and interdisciplinary colleagues over the last decade at the University of Memphis, McGill University, Illinois Institute of Technology, North Carolina State University, and the University of Central Florida. Over the years, the design of the STEM content has included experts in several fields of STEM and biomedical sciences. Figure 1 illustrates MetaTutor’s main interface elements.

**FIGURE 2:**
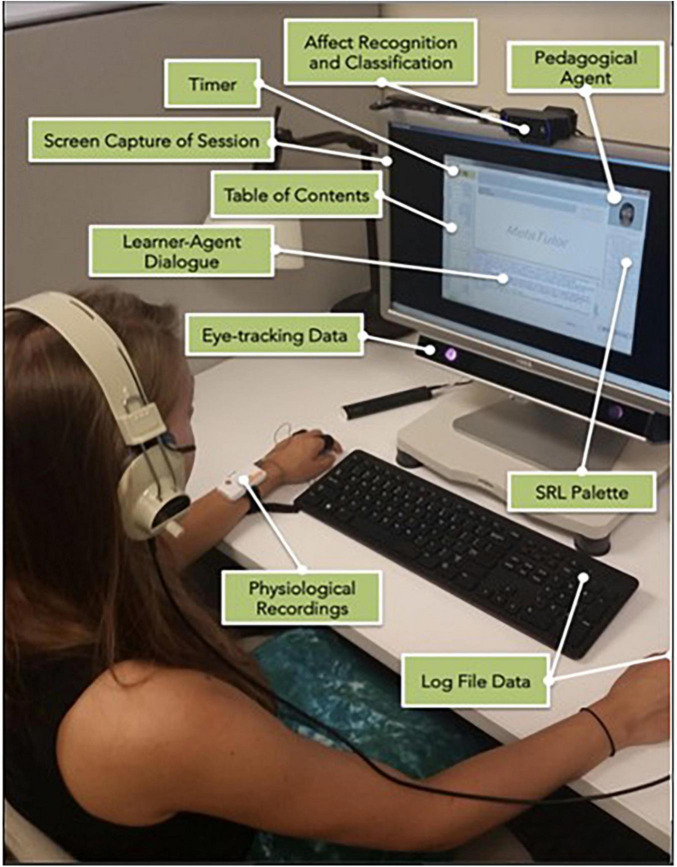
Instrumented student participating in a typical MetaTutor study.

**FIGURE 3:**
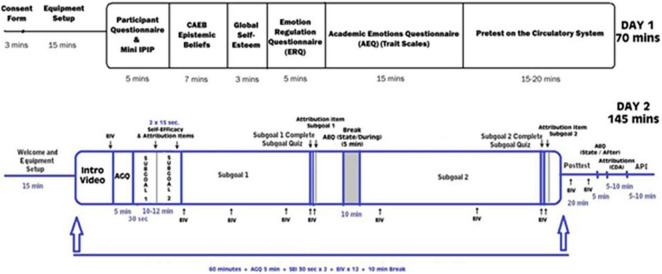
Experimental procedure used for all MetaTutor studies.

MetaTutor is aligned with theoretical, conceptual, and methodological assumptions about SRL and learning with advanced learning technologies (Winne and Hadwin, 1998, 2008; Pintrich, 2004; Azevedo, 2005; Azevedo et al., 2010, 2019; Zimmerman, 2011; Schunk and Greene, 2018; see Figure 1). First, CAMM processes can be detected, tracked, and modeled using online trace methodologies. Second, students deploy these processes during extended interactions with MetaTutor while instrumented and participating in our laboratory experiments (see Figure 2 for experimental set-up). Third, the CAMM signatures collected from the various methods, techniques, devices, and sensors (e.g., facial expressions of emotion, physiological sensors, eye tracker, log files, and screen recording of student-system interactions) will have different profiles depending on real-time fluctuations in response to internal and external conditions (e.g., accumulating knowledge about the topic and feedback from the PAs, phases of learning, or generation of subgoals. Fourth, a session is characterized by learner-generated subgoals). Fifth, several types of trace, self-report, and product data are identified as critical for examining the complex nature of SRL. In our studies, trace data included think-alouds, eye tracking, log files, and physiological recordings. Product data represented three individual pretest measures that assessed different types of knowledge including declarative, procedural, and mental models; equivalent measures were also given as a posttest. We included self-report measures of motivation and emotions that were also presented at pretest, during learning, and at posttest. Figure 3 illustrates the experimental procedure for all MetaTutor studies.

To experimentally test the effectiveness of the scaffolding provided through the system, MetaTutor features two experimental conditions, i.e., adaptive scaffolding and no scaffolding. In the former condition, PAs prompt students to engage in several learning strategies (e.g., prior knowledge activation, note-taking, or judging the relevance of a page to the current learning sub-goal) based on the student’s interaction with the system (e.g., the goals they set, how much time they spent with certain contents). Further, students receive feedback for prompted or self-initiated assessments, such as quizzes. In the no scaffolding condition, no such prompts or feedback are provided. However, students are free to use any of the learning strategies incorporated in MetaTutor (see SRL palette on the right-hand side of Figure 1) but do not receive feedback regarding these interactions.

## MetaTutor’s Architecture

**FIGURE 4:**
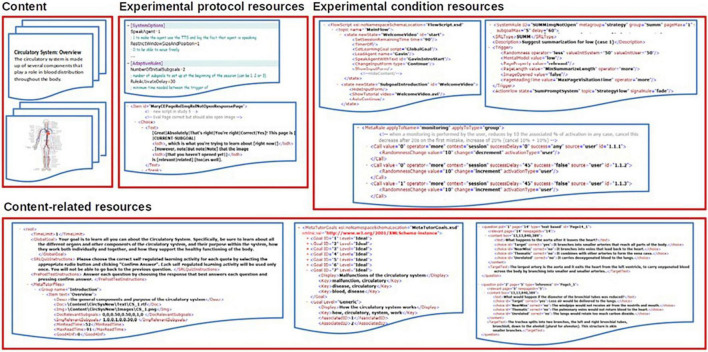
MetaTutor’s overall architecture.

MetaTutor’s architecture relies on the use of three types of external resources (see Figure 4 for the overall architecture): (1) content and content-related resources, (2) experimental protocol resources and, (3) experimental condition resources.

The content resources include the pedagogical material on the circulatory system provided by 48.RTF files and as many.JPG images (one per page), which are displayed at the center of the interface while the student is learning with MetaTutor (see Figure 1 for the interface overview). Three additional XML files help in structuring the content: (a) a file is used to structure the table of contents sections and subsections and to associate to each page the RTF and JPG files as well as the subgoals associated to the page, and a minimum and maximum reading times (estimated from a sample of students who read the content outside of MetaTutor), (b) a file is used to define the 7 subgoals existing in the usual version of MetaTutor and to associate to each of them a set of keywords used during the interactive subgoal setting phase at the beginning of the session and anytime a student has validated all their initial subgoals, (c) a file is used to define the questions associated to each content page (6 questions per page: 3 based on the text, 3 requiring an inference from the student) with 4 possible options (the correct answer, a “near miss” corresponding to a wrong answer close from the correct one, an incorrect answer that is related to the question and an incorrect answer that is completely unrelated)—this file is used to dynamically generate the quizzes associated to each page by randomly drawing 3 questions from amongst them. MetaTutor’s seven sub-goals include the path of blood flow, how does the heartbeat, what are the functions of the components of the heart, what are the functions of the components of the blood vessels, what are the functions of the components of blood, purposes of the human circulatory system, and malfunctions of the human circulatory system.

The experimental protocol resources are used to change some overall parameters of MetaTutor to adapt to particular experiment settings. It includes a text file made of a set of attribute-values defining parameters such as whether the agent should speak or not, the name and number of the experimental conditions, the number of subgoals to set initially, the minimum time before a PA can intervene on a page, etc. The second experimental protocol file is an XML file defining the scripts associated with the PAs, such as what text is displayed in the dialog history, what is actually said by the agent with possible variations to avoid repetition. Specifically, a given script can have several different texts associated with it, and within a text, regular expressions add more variability. It is also possible to use special tags to ensure that some additional details are provided the first time an agent says this text but not later times. Finally, the experimental condition files are a set of three files for each of the experimental conditions tested (i.e., 9 files overall if there are three conditions). The first file defines as a finite state automaton the overall flow followed by MetaTutor when a student is assigned to that experimental condition. For each state, it can define, through a set of predefined tags, which PA to show, what script that agent should say and display, how to change the system interface (e.g., to show the summary interface or a self-report questionnaire), whether to pause or resume the system, etc. and the next state depending on the student’s actions. It allows MetaTutor to alternate between guided phases (at the beginning and end of the learning session and every time the student engages in an SRL process) and phases where the student can freely explore the pedagogical content. The second file defines both the actions to trigger when using the SRL palette (see Figure 1), each PA’s intervention, and what can trigger these actions. Each intervention is associated with a set of predefined conditions: (1) the student has spent more than the average reading time on a page, (2) that page is relevant to the student’s current subgoal, (3) the student has not done more than a predefined number of content evaluations (CEs) while working on this subgoal, and (4) the student has not evaluated the relevance of the content of this page already. When conditions are met, there is a probability that a PA will intervene to ask them to monitor their learning or to deploy a learning strategy (e.g., asking them to evaluate the relevance of this content to their subgoal). Finally, the third file defines a set of meta-rules that can modify the triggering conditions of the PA’s interventions, thus making the rules dynamic over the learning session based on the student’s overall use of SRL processes. For instance, if a student tends to regularly assess how relevant the content of the page, they read is to their current subgoal, the associated PA’s intervention will be less likely to be triggered on a page by adjusting the probability parameter of the rule. These meta-rules allow, for instance, to define a more intense prompting at the beginning of the session which will gradually decrease if the student performs the SRL strategies correctly, and even faster if they initiate them (Bouchet et al., 2016).

In the original version, four separate PAs embodying four different functions (guiding through the system, planning, monitoring, and using learning strategies) provide verbal feedback and engage in a tutorial dialogue to scaffold students’ selection of relevant subgoals based on their level of understanding of the circulatory system, accuracy of metacognitive judgments, and use of learning strategies. Each of the four PAs had a different function based on SRL. One of the four PAs (Gavin, Pam, Mary, or Sam) is always displayed in the upper right-hand corner of the environment (see Figure 1). These agents provide varying degrees of adaptive scaffolding (i.e., prompting and feedback) throughout the learning session to scaffold students’ SRL skills such as summarizing, making JOLs, and understanding content (see Azevedo et al., 2010 for details). Briefly, each agent serves a different purpose: (1) *Gavin the Guide* helps students to navigate through the system and orient the students about the task; (2) *Pam the Planner* guides students in setting appropriate sub-goals by activating their prior knowledge and coordinating sub-goals; (3) *Mary the Monitor* helps students to monitor their progress toward achieving their sub-goals by prompting and scaffolding several metacognitive processes such as FOKs, JOLs, and CEs; and (4) *Sam the Strategizer* helps students deploy SRL learning strategies, such as summarizing and note-taking, making inferences, re-reading, and generating hypotheses. Learners can interact with these PAs and enact specific SRL learning processes by selecting any feature of the SRL palette displayed at the right-hand side of the interface during the learning session.

For example, students are prompted to self-assess their understanding and are then given a brief quiz. Quiz results allow the PA to provide feedback according to the calibration between students’ confidence of comprehension and their actual quiz performance. Learners can also self-initiate and express these same system-initiated metacognitive judgments and learning strategies through an SRL palette of actions (see Figure 1). For example, they can click a button to indicate they want to make a statement about their understanding of a page and then indicate on a scale that their understanding is poor. They can also indicate that they want to summarize the content of that page and type their summary in a textbox. MetaTutor collects information from user interactions to provide adaptive feedback on deploying SRL behaviors.

In the next sections, we describe our findings based on their contributions to cognitive, metacognitive, affective, and motivation processes underlying learning with MetaTutor. Please note that although we strive for consistency in structure, each section differs slightly given the number of published studies, specific research foci, and research questions that were answered based on the specific CAMM SRL process.

## Role of Cognitive Strategies During Learning With MetaTutor

Cognitive processes that are carried out in the service of studying and learning involve processing new information in order to transform it into long-term memory (Winne, 2018). In learning environments, such new information is predominantly represented by multimedia formats, including texts, graphics, and audio (Mayer and Fiorella, in press). Cognitive processes are applied to these new inputs to create mental representations for each modality and to form connections between new inputs and prior knowledge already stored in long-term memory, an underlying phenomenon described by Winne and Hadwin’s (1998; 2008) IPT of SRL. These processes are critical during learning about complex science materials as their use allows learners to transform instructional materials into knowledge structures that change over time during interactions with systems such as MetaTutor. For example, learners can summarize instructional content or make hypotheses about blood flow after reading and inspecting relevant diagrams. Given the overall objective for MetaTutor to scaffold effective SRL processes, the design of this ITS was informed by Winne and Hadwin’s (1998; 2008) IPT model of SRL to help scaffold and support cognitive operations.

Cognitive operations are the processes that transform external information into mental representations and other learning products, such as notes or essays and new mental models of how the human circulatory system works. Winne (2001) further specifies cognitive operations into several smaller grained processes: searching, monitoring, assembling, rehearsing, and translating (SMART; Winne, 2018). These specific cognitive operations, when applied individually or in combination during learning, may account for a variety of study tactics and learning strategies during the third phase (enactment) of SRL, including reading or re-reading, integrating texts and graphics and transforming information across modalities, taking notes or writing summaries, making inferences, memorizing, or elaborating. Empirical investigations of MetaTutor have largely focused on note-taking as one such cognitive learning strategy.

### Note-Taking

Note-taking is a prevalent cognitive learning strategy that allows students to “record, clarify, organize, and comprehend information” (Bonner and Holliday, 2006, p. 787; Lee et al., 2013). Depending on the quality of the execution, taking notes may support the integration of new information and the construction of a coherent mental representation of the instructional content. Beyond creating a product that may be viewed and studied later, the process of taking notes may itself be beneficial for learning (Kiewra, 1985; Morehead et al., 2019). In sum, notes taken during studying or learning provide a perspective on the cognitive processes enacted during this phase that may be relevant for successful learning.

Trevors et al. (2014) directly examined the quantity and quality of college students’ notes as they learned with MetaTutor. They sought to determine whether these note-taking variables and their predictive relationship to subsequent learning varied as a function of note-takers’ prior knowledge and the experimental condition to which they were assigned (i.e., prompt and feedback vs. control). To evaluate the quality of notes, Trevors et al. (2014) coded whether conceptual phrases in notes represented either a *deep* or *shallow* reflection of the instructional concepts that students were studying at the time of creation. A deep representation in notes signified that students went beyond the information presented in the instructional content to include new information or identify connections or themes across instructional texts and diagrams or between instructional content and prior knowledge. Such elaboration is thought to reflect the learner’s comprehensive understanding of the underlying relevance and meaning of the instructional content beyond what is explicitly stated. Conversely, a shallow representation in notes signified a simple verbatim reproduction of the instructional content that is consistent with rote memorization or rehearsal strategies and a superficial understanding of the content. As quantitative properties of notes, the frequency and duration of note-taking episodes and the number of conceptual phrases were also examined. Findings showed that in this context, notes were largely shallow verbatim copies of instructional content that in turn negatively predicted learning. Students with low prior knowledge spent more time on this counterproductive learning strategy compared to their high prior knowledge counterparts, which suggests that the low prior knowledge group may have over-relied on a knowledge-building strategy at the expense of monitoring its effectiveness. MetaTutor system prompts and feedback substituted other SRL processes in lieu of note-taking, which was significantly lower in the experimental condition compared to the control.

In a subsequent study, Taub and Azevedo (2019) adopted different methodological and analytical approaches to studying the interrelationships between prior knowledge and note-taking within MetaTutor. In addition to mining computer log files of user interactions, Taub and Azevedo analyzed sequences of several cognitive processes together. Further, they employed eye tracking to see what instructional content and features learners were attending to on the system interface and patterns of eye gaze across these areas. They found that high prior knowledge learners engaged in sequences of learning strategies that involved note-taking or summarization more than their low prior knowledge counterparts. Consistent with this finding, Taub and Azevedo (2019) also found that high prior knowledge learners had greater frequencies of fixating on pairs of areas of interest (AOIs) that showed attention to the instructional text and the note-taking interface than their low prior knowledge counterparts (e.g., fixated more on the instructional text followed by fixating on the note-taking interface). These findings suggest that high prior knowledge students were able to cycle between content and cognitive strategies involving note-taking quickly and fluidly.

More recently, Wiedbusch et al. (2021b) examined why learners lack sufficient SRL skills to successfully implement strategies (e.g., JOL, note-taking, self-testing, etc.). The authors used principal component analysis (PCA) on log files to further explore underlying patterns in the frequency of strategy deployment occurring with and without PA scaffolding. The motivation for this study was to use a data-driven approach to find underlying structures of the system- and learner-initiated cognitive and metacognitive SRL strategy use. This study provided empirical evidence that the system’s underlying architecture deployed cognitive and metacognitive processes corresponding to both the phases of learning according to Winne’s (2018) theory of SRL, the familiarity of processes, and type of effort allocation. The authors highlight the potential to incorporate quality of SRL strategy use (e.g., such as the quality of note-taking measured in Trevors et al., 2014) in future work to reveal how standards (as defined in the Winne (2018) theory impact student strategy deployment. Future iterations of the design of these skills could also then incorporate quality in the conditional procedural rules.

### Goal-Setting

Setting goals is a critical part of the second phase of Winne and Hadwin’s model, yet little research has examined how we set goals and how we might do so collaboratively with a PA. Harley et al. (2018) contributed to addressing this gap in the literature by drawing on theories of co- and socially shared regulated learning (Järvelä and Hadwin, 2013) to identify patterns in learner-PA interaction and, including students’ compliance with the PAs’ suggestions, subsequent associations with learning outcomes. Learner-PA interactions were examined across two scaffolding conditions: adaptive scaffolding and no scaffolding. Learners’ compliance to follow the PA’s prompts and feedback in the adaptive scaffolding condition were also examined. Results demonstrated that learners followed the PA’s prompts and feedback to help them set more appropriate subgoals for their learning session the majority of the time. Descriptive statistics revealed that when subgoals were set collaboratively between learners and the PA, they generally lead to higher proportional learning gains. Taken together, the results provide preliminary evidence that learners are both willing to engage in and benefit from collaborative interactions with PAs when immediate, directional feedback and the opportunity to try again are provided.

### Lessons Learned and Future Directions

Findings from Harley et al. (2018) have implications for extending co- and socially shared regulated learning theories to include learner-PA interactions, rather than just learner-learner and learner-teacher. Findings from Taub and Azevedo (2019) suggested that learners with high prior knowledge engaged in more sequences of actions including note-taking or summarization, in contrast with findings from Trevors et al. (2014), where learners with high prior knowledge took fewer notes overall. The conflicting results may be attributable to differences between sample characteristics or changes in system versions of MetaTutor across time. However, two other salient differences between studies are the different methodological and analytical approaches used. In particular, methodologically, Taub and Azevedo used eye-tracking, which provides high temporal resolution in assessing attention allocation across studying that also allows for inferences regarding cognitive strategies. Analytically, while Trevors et al. (2014) examined individual instances of specific quantitative and qualitative variables of notes directly, Taub and Azevedo (2019) focused on patterns and sequences of learning processes rather than aggregated events. Together, these research design choices—namely, the direct observations for coding, temporal specificity, and contextual sequence in which a cognitive learning process is enacted—provide a different perspective on the same phenomenon.

Working with multimodal multichannel data to assess a variety of learning strategies and their sequences presents many different ways to study the same construct, which is a key future direction highlighted by Wiedbusch et al. (2021a). As researchers’ technological capacity to collect cognitive process data grows, so too must they develop analytical frameworks to keep pace (Azevedo and Gašević, 2019; Järvelä and Bannert, 2019). A challenge for researchers will be to arrive at some consensus regarding several standardized operationalizations or, at minimum, an explicit understanding of what different channels may and may not reveal about cognitive learning strategies and ultimately learning outcomes. We argue that a multimodal learning analytic (MLA) approach could be suitable for this kind of data. MLA uses data from different sources about learning traces for doing a single analysis, finding how to combine, or fuse, the data extracted from several sources/modalities in order to provide a more comprehensive view of learning processes. To date, individual events within one channel have been integrated to assess sequences and multiple channels have been conceptually integrated into the discussion. However, true analytical integration will entail the fusing of data channels (e.g., log files, eye tracking, and think-alouds) into quantifiable units appropriate for statistical analyses. This would enable more objective, complete and valid measurements of complex cognitive processes that manifest as study tactics or multiple tactics organized into learning strategies. The need for integration and valid measurement will only grow as teaching and learning become increasingly mediated via new immersive technologies such as augmented or virtual reality and user interfaces such as ITSs.

## Role of Metacognition During Learning With MetaTutor

Metacognition plays a key role in monitoring several aspects of oneself, task, learning situation, and context (Nelson and Narens, 1990; Efklides et al., 2018; Schunk and Greene, 2018; Winne, 2018; Winne and Azevedo, 2022). Accordingly, we expect students to dynamically and accurately monitor and regulate their cognitive strategies while using MetaTutor to learn about the human circulatory system. While metacognitive processes are ideally captured using trace methods such as concurrent think-aloud protocols (Azevedo and Cromley, 2004; Greene and Azevedo, 2009; Greene et al., 2021), during a MetaTutor learning session, metacognitive processes can either be prompted by the PA, or students can self-initiate the use of the same metacognitive processes via the SRL Palette (see Figure 1). The SRL Palette allows students to judge how well they understand the content they are currently reading (JOL), rate how familiar they are with the content they are currently reading (FOK), and assess how relevant the content text and diagram are for accomplishing their current sub-goal (CE). Students can also indicate they have read enough content pages to complete their sub-goal (monitoring progress toward goals; MPTG). We highlight that these are only four possible metacognitive judgments (Nelson and Narens, 1990; Koriat, 2015; Dunlosky and Rawson, 2019) and that students may have monitored themselves (e.g., regulated their emotions to address misunderstanding content; McRae and Gross, 2020), the task, learning situation, and context using other metacognitive processes that were not captured since our studies relied on student-PA interactions and the SRL Palette.

When investigating metacognitive processes in MetaTutor, there have been three main categories of research questions. First, *what* are the factors that impact the use of metacognitive processes during learning with MetaTutor? Second, *when* do students engage in metacognitive processes during learning with MetaTutor? Third, *how* or *how accurate* are students when engaging in metacognitive processes during learning with MetaTutor?

### Which Factors Impact the Use of Metacognitive Processes?

A first study relied on three clusters of students created from features extracted from the log files only (Bouchet et al., 2013), using differential sequential mining (Bouchet et al., 2012) to see what sequence of actions differentiated high and low performing students. It revealed that high-performing students tended to be better at quickly identifying the relevance of a page to their subgoal, were more methodical in their exploration of the pedagogical content, relying on system prompts to take notes and summarize, and were more strategic in their preparation for the post-test (e.g., using the end of their session to briefly review pages).

**FIGURE 5:**
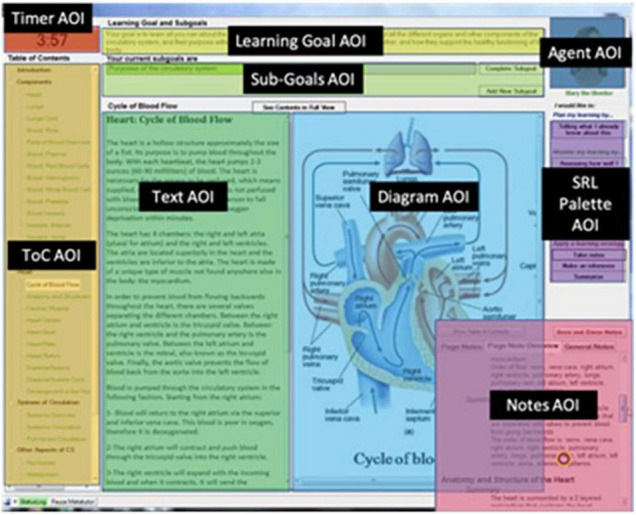
Areas of interest on MetaTutor’s main interface.

Further studies have investigated differences in the frequency and duration of using JOLs, FOKs, CEs, and MPTGs between groups, such as high vs. low prior knowledge (Taub et al., 2014; Taub and Azevedo, 2019), and the experimental compared to the control condition (Azevedo et al., 2011, Azevedo et al., 2016a). To investigate the impact of the use of metacognitive processes by prior knowledge group, Taub et al. (2014) and Taub and Azevedo (2019) used log files only, or eye tracking and log files, respectively, to examine how students with high or low prior knowledge engaged in frequencies of JOLs, FOKs, CEs, and MPTGs during learning with MetaTutor. Prior knowledge was defined by conducting a median split on pre-test score (score on a 30-item multiple-choice content test about the circulatory system). Results revealed that students with high prior knowledge engaged in higher frequencies of JOLs and MPTGs, and lower frequencies of FOKs and CEs than students with low prior knowledge (Taub et al., 2014). In a different study, results from eye-tracking data revealed no differences in the number of fixations on AOIs related to engaging in metacognitive (and cognitive) processes, however, there were significant differences in frequencies of engaging in AOI-pairs; i.e., fixating from the text content to one of eight other AOIs on MetaTutor’s main interface (see Figure 5) between prior knowledge groups (Taub and Azevedo, 2019). Specifically, students with high prior knowledge engaged in significantly higher frequencies of AOI-pairs than students with low prior knowledge.

A potential interpretation of results from both studies indicates students with different levels of prior knowledge allocate resources differently for engaging in metacognitive processes during learning with MetaTutor. However, results are inconclusive because results in Taub et al. (2014) indicated a higher frequency of engaging in total metacognitive processes, but not for all micro-level metacognitive processes. Additionally, when examining total fixation duration, there were no significant differences, but there were differences when examining frequencies of engaging in fixation pairs. Thus, depending on the level of granularity (i.e., micro-level processes, single fixation vs. fixation pairs), results may be inconsistent with each other.

Another factor that has been found to impact the use of metacognitive processes during learning with MetaTutor is experimental condition. A previous study examined differences in the use of both metacognitive and cognitive processes between the prompt and feedback and control conditions during learning with MetaTutor (Azevedo et al., 2016a,b). This study examined how students self-initiated the use of these processes by clicking on the SRL palette. Results revealed that students who were provided with adaptive scaffolding (i.e., in the prompt and feedback condition) engaged in significantly more JOLs and CEs than students in the control condition (after controlling for pre-test score), and although not significant, frequencies of engaging in FOKs and MPTGs were also higher for students in the adaptive scaffolding condition. This demonstrates the beneficial effects of providing scaffolding to students as external regulation because these processes were self-initiated, and so even though students were prompted to engage in metacognitive processes, this influenced how they self-initiated the use of these processes as well. These findings show how both self- and external regulation can have beneficial effects on using metacognitive processes during learning with MetaTutor. The above-mentioned studies provide evidence for how there are different factors that have been found to impact how students use metacognitive processes during learning with MetaTutor.

### When Do Students Engage in Metacognitive Processes?

According to theories of SRL, metacognition can occur before, during, or after a cognitive process (Winne and Azevedo, 2014, in press). In the previously mentioned studies (Taub et al., 2014; Taub and Azevedo, 2019), additional analyses used educational data mining techniques to investigate sequences of metacognitive (and cognitive) processes during learning. One study used log files to investigate quintet sequences of engaging in metacognitive (and cognitive) processes (Taub et al., 2014). Results revealed that it was more common for students with low prior knowledge to engage in metacognitive processes at the end of the quintet sequence, whereas students with high prior knowledge engaged in metacognitive processes in the middle of the sequence. In addition, this study examined the use of metacognitive (and cognitive) processes by sub-goal, and results revealed that students with both levels of prior knowledge engaged in different numbers of processes for different sub-goals, where students engaged in more processes when working on a more difficult (categorized based on the content included) sub-goal.

Taub and Azevedo (2019) used sequential pattern mining and differential sequence mining to examine patterns via log files of engaging in metacognitive and cognitive processes. Results revealed students with high prior knowledge engaged in sequences that contained both cognitive and metacognitive processes, and students with low prior knowledge engaged in sequences with metacognitive processes only. Additionally, the only sequence frequency that was higher for low prior knowledge students contained inaccurate metacognitive processes.

More recently, Dever et al. (2021) examined how undergraduate students engaged in self-initiated and system-facilitated self- and externally regulated micro-level metacognitive processes (i.e., CEs, JOLs, FOKs, and MPTGs) to process MetaTutor’s science content. This study explored the relationship between students’ average monitoring micro-process strategy frequencies and learning gains through a person-centered approach as students interacted with MetaTutor. Using hierarchical clustering, Dever et al. (2021) found that clusters differing in metacognitive monitoring process usage had a significant difference in their learning gains where students who used a greater proportion of CEs and FOKs had greater learning gains than learners who used greater MPTG strategies. These aforementioned studies demonstrate differences of when students engage in metacognitive processes during learning with MetaTutor. Specifically, levels of prior knowledge contribute to students’ differences in their deployment of cognitive and metacognitive processes and strategies.

### How Accurate Are Students at Deploying Metacognitive Processes?

In addition to examining the sequences of engaging in metacognitive processes, studies have also investigated the *quality* of making metacognitive judgments and engaging in monitoring processes during learning with MetaTutor, and what has been found to impact these judgments (Feyzi-Behnagh et al., 2011; Taub et al., 2018, 2021). For example, Feyzi-Behnagh et al. (2011) investigated the impact of three different conditions (prompt and feedback, prompt only, or control) on students’ metacognitive judgments during learning with MetaTutor. They used log files to analyze the relationship between students’ judgments for JOLs and FOKs (with + and—valences) and subsequent quiz performance. Results revealed that in general, students were fairly inaccurate at making JOLs and FOKs, and were fairly over-confident when making these judgments, especially in the prompt only condition. Thus, their results provided strong evidence for providing students with prompts *and* feedback for providing effective scaffolding to students during learning with MetaTutor.

Studies have also examined the accuracy of metacognitive processes, and how this has been impacted by emotions or affective states using log files and facial expressions. Taub et al. (2021) examined the relationship between evidence scores of emotions and the accuracy of metacognitive and cognitive judgments. Results found mean evidence scores of surprise negatively predicted accuracy of making FOKs (and mean evidence scores of frustration positively predicted accuracy of notes). In another study, Taub et al. (2018) also used log files and videos of facial expressions and examined the interaction between evidence of action unit (AU) 4 (eyebrow lowerer) and prior knowledge, and how they impacted the accuracy of JOLs, FOK, CEs, and MPTGs. They investigated each instance of engaging in a metacognitive process using multilevel modeling, and results found accuracy was highest for students with high prior knowledge and low levels of AU4. However, for students with low prior knowledge, accuracy was highest with high levels of AU4, demonstrating the differential impacts of emotional states on the use of metacognitive processes. These three example studies exhibit how there are different factors that have been found to impact the accuracy of engaging in metacognitive processes during learning with MetaTutor.

### Lessons Learned and Future Directions

Based on the numerous studies that have investigated the use of metacognitive processes during learning with MetaTutor, there are many take-away lessons that can be used toward assessing metacognition and developing advanced learning technologies that foster the effective use of these processes, from a theoretical and empirical perspective.

First, in line with Winne and Azevedo (2014, in press) who defined the timing of using metacognitive processes in relation to cognitive processes, it seems that to successfully examine all the components of using metacognitive processes (i.e., what, when, why, and how), we should examine both cognitive and metacognitive processes simultaneously. Prior studies have shown that students do engage in both of these processes together during learning with MetaTutor, and it is important to consider when in a sequence (if done so) one precedes or follows the other. In addition, extending these analyses to include affective and motivational processes as well (see sections below) will provide even more contextual information regarding the use of these processes (as seen in Taub et al., 2018, 2021). Without adding the affective component, it would be unclear that prior knowledge can impact metacognitive monitoring differently with different levels of expressing action unit (AU) 4.

Future studies should examine how motivation impacts engaging in metacognitive processes as well. Cloude et al. (2018) examined how goal orientation (categorized into separate groups for mastery/performance/combination of the 2, and approach/avoidance/combination of the 2) impacts the frequency of using metacognitive processes during learning with MetaTutor. However, there were no significant differences between groups. It is possible that administering the achievement goal questionnaire once was not able to capture a complete and dynamic measurement of goal orientation, and perhaps we can detect motivational differences and how it impacts metacognition by exploring a new methodology for measuring motivation, such as electrodermal activity or changes in affective states (Winne and Perry, 2000; Zimmerman, 2011). Additionally, studies demonstrated the need to use different theoretical frameworks for different research questions investigating metacognitive processes. As an implication of this, a unifying framework for metacognition should be developed to address all components of research that can be conducted to examine metacognition.

Results demonstrate that in different situations or given different contextual factors, learners might benefit from using metacognitive processes in different ways. By knowing this, do we want to continue randomly assigning them to conditions we know will not be useful to them? For example, students with low prior knowledge demonstrated a lack of use of cognitive strategies in Taub and Azevedo (2019), but do we want to inundate them with prompts when they need to allocate a substantial number of resources to learn the material? If students (regardless of prior knowledge) are not being provided with any feedback from the PA from being in the control condition, are they at a disadvantage? How does this impact how we design experimental conditions to ensure sufficient randomization? Thus, implications for future research that examines learning with advanced learning technologies should encourage researchers to employ a within-subjects design to ensure students are exposed to all possible learning contexts so they can benefit from learning with these systems. Additionally, we should consider how a learner’s needs may change over the learning session (i.e., they may require prompt and feedback support early, but not later on) and over multiple learning sessions as they learn more strategies.

Finally, the abovementioned studies demonstrate the usefulness of using multimodal multichannel data to investigate metacognitive processes during learning with MetaTutor (Azevedo et al., 2018, 2019). These studies predominantly used log files, but also eye tracking and videos of facial expressions. Using more data channels provides greater insight into how students engage in metacognitive processes (in terms of the *what, when*, and *how*), and how metacognition interacts with cognitive, affective, and motivational processes when investigating self-regulatory behaviors during learning with MetaTutor.

## Role of Emotions During Learning With MetaTutor

Emotions play a critical role in SRL as they can impede and interfere with learning if not monitored and regulated dynamically and accurately during learning across tasks, contexts, and with advanced learning technologies such as MetaTutor (D’Mello and Graesser, 2012; Pekrun and Linnenbrink-Garcia, 2014; Efklides et al., 2018; McRae and Gross, 2020). The descriptive and correlational studies described in this section aimed to discover what kinds of emotions learners experienced while interacting with MetaTutor. More specifically, studies aimed to describe how emotions changed over time, associations between individual differences (e.g., trait emotions and personality traits), the alignment of different emotional expression components, and corresponding methodologies (automatic facial recognition software, skin conductance sensors, and self-reports), and emotions directed toward different virtual PAs. In order to accomplish these objectives, we drew on the control-value theory (CVT) of achievement emotions (Pekrun, 2006; Pekrun et al., 2011).

CVT was selected because it provided us with more detailed propositions than Winne and Hadwin’s (2008) theory of SRL to guide the formulation of research questions and hypotheses as well as methodological decisions specific to emotions (compared to questions about cognitive and metacognitive processes used in many MetaTutor studies). Our research drew on the operational definition of achievement emotions advanced in the CVT that emotions can be characterized by valence, activation, object focus, and time frame. Valence refers to the pleasantness (i.e., positive valence; e.g., enjoyment, hope) or unpleasantness (i.e., negative valence; e.g., frustration, anxiety) of an emotion. Activation corresponds to the degree of physiological activation (i.e., arousal; Russell et al., 1989). In addition, achievement emotions can arise from a focus on either an achievement activity or an outcome (object focus). Boredom from studying a chapter is an example of an activity emotion, whereas anger about one’s low score on an exam is an example of an outcome emotion. The time frame can be prospective (future-oriented), concurrent (present moment), or retrospective (past-oriented). The emotions that are elicited from recalling how one did on a test are retrospective emotions because they involve thinking about success or failure that has already occurred (e.g., joy or frustration). Prospective emotions, on the other hand, are emotions related to future activities and outcomes, for example, experiencing anxiety while thinking about one’s potential grade on an exam one does not feel prepared to take. Concurrent emotions include emotions aroused from an activity one is currently undertaking, such as enjoyment or boredom during a lecture. The CVT also assumes that emotions have multiple expression components including experiential, behavioral, and physiological activation.

### Synthesis of Key Findings From Published Studies

Our first question concerning emotions in MetaTutor was the incidence of different emotions. A deceptively simple question that has layers we endeavored to tackle. The first general layer was a temporal one: How is the time period that emotional occurrence is evaluated and defined, and how stable are emotions over time? A second layer was: Which emotion expression component is supplying us with the data we are using to answer our question and does using different channels provide us with a different answer? We developed a single-item self-report measure to assess 19 different concurrent state emotions and help explore these lines of inquiry: The emotion-value (EV) questionnaire (Harley et al., 2013, 2015). The EV questionnaire was administered on five occasions during a study with MetaTutor which provided us with five snapshots of learners’ present in-the-moment emotional experiences. By asking learners how they felt “right now” we were also able to align data from other emotion expression channels to assess agreement between self-report, behavioral, and physiological expression components.

#### What Kinds of Emotions Did Learners Tend to Experience While Learning With MetaTutor and Did These States Change Over the Course of Their Learning Session?

In our first article using the EV and automatic facial expression recognition software (FaceReader 5), we found that neutral and positively valenced activating emotional states represented the majority of emotional states experienced with MetaTutor across channels (Harley et al., 2013). The low incidence of negative emotions was favorable, especially considering that MetaTutor did not employ gamification features (e.g., story elements; Harley et al., 2016a,b) to enhance enjoyment, nor was the content designed to be related to students’ academic degree. The latter was expected to result in lower appraisals of task value, which can dampen the intensity of both positive and negative emotions (Pekrun, 2006). It is also worth noting that when using the meta-rules to trigger a more intense initial prompting from the PAs, we noticed an increase in frustration (and sometimes boredom), as well as a significantly higher level of confusion in low prior knowledge students compared to high prior knowledge students—which is consistent with the fact that high prior knowledge students are better at self-regulating their learning (Bouchet et al., 2018). Moreover, examining the PAs-directed emotions revealed the importance of feedback to maintain negative emotions at a low level, as a prompt-only condition tended to trigger more negative emotions (such as anger) than a prompt-and-feedback one (Harley et al., 2011). These negative agent-directive emotions are key to monitoring as although they do not affect the use of SRL processes, they were significantly related to negative learning gains (Mudrick et al., 2014). They seem partly related to the perceived competency of agents, as the least liked PA used to encourage students to deploy learning strategies was shown to negatively impact students’ experience of enjoyment and the frequency of his interventions predicted their report of boredom while using MetaTutor (Mudrick et al., 2015). Analyzing facial expressions over some particular phases of interactions with MetaTutor such as the subgoal setting phase also confirmed the importance of considering the notion of co-occurring emotions (Conati and Maclaren, 2009), as nearly a quarter of students’ embodied emotions were co-occurring ones (Harley et al., 2012).

Self-report results from this study (Harley et al., 2013) also revealed statistically significant changes in emotions over time, most often, a decline in levels of positively valenced and neutral states across the learning session. Facial expression results revealed that most learners were classified as being in a neutral state at each of the five 10-s windows before the administration of the EV where facial expression data was drawn from to align with the self-report data. In looking at transitions between the five time points, most transitions away from neutral were toward happiness (the only positively valenced emotion FaceReader classifies). Those learners who expressed a negative emotion tended not to remain fixed in that state.

Results from self-report and facial expression channels appeared to tell a different story. Self-reported emotions revealed a decline in levels of positively valenced and neutral emotional states and an increase in some negative, activating emotions (e.g., frustration) that might call for interventions to sustain positive and neutral states. On the other hand, facial expression recognition data suggested that emotional states were relatively stable and that most transitions were relatively short-lived and between positive and neutral states. These apparently conflicting results highlight the benefit of collecting data from different channels. In this case, self-report measures were more sensitive to different levels of intensity (i.e., endorsement) as well as a broader variety of emotional states (19 states vs. seven) compared to facial expression recognition software. As such, we interpreted the emotional dynamics from this study as complementary, with the EV results showcasing more granular patterns than those observed with the facial expression recognition software (Harley et al., 2013).

Another study by Cloude et al. (2020) captured and analyzed 117 college students’ concurrent and self-reported negative emotions across 3 time points during learning with MetaTutor using D’Mello and Graesser (2012) model of affective dynamics: (1) confusion, (2) frustration, and (3) boredom. They found that when increases in boredom occurred across the three time points, it was related to learners initiating less accurate metacognitive monitoring processes and less learning of the circulatory system after the session. Results also suggested that when confusion persisted for too long over the time points during learning, it was related to less learning after the session (Cloude et al., 2020).

To investigate this finding more deeply, Cloude et al. (2021a,b) studied the relation between emotional dynamics and its impact on cognition and learning with MetaTutor by multiple components using Plass and Kaplan’s (2016) Cognitive-Affective Model of Multimedia learning and Russell (1980) Circumplex Model of Affect. Emotions were defined by (1) temporality (i.e., increase, decrease, or no change), (2) valence, and (3) activation across six data points from 174 undergraduates’ self-reported emotions. Latent growth models were calculated and revealed that the stability of negative activating emotions over time was negatively related to performance while controlling for prior knowledge and that changes in negative deactivating emotions were negatively related to time spent engaging in cognitive strategies during learning activities. Finally, a random forest classifier revealed high accuracy in predicting high (top 30%) and low-performance groups (bottom 30%) using pre-test scores, changes in negative deactivating emotions, and time engaging in cognitive strategies. These findings have important implications for designing affect-aware systems that can potentially leverage emotion interventions based on if, when, and how an emotion changed (or remained stable) to optimize time engaging in cognitive strategies and performance outcomes with emerging technologies (Harley et al., 2017; Cloude et al., 2021a).

#### Were Different Emotional Expression Components Tightly or Loosely Coupled?

In order to examine the level of agreement (i.e., coupling) between emotional expression components we extended our analyses with self-report and facial expression recognition software from Harley et al. (2013) to include skin conductance level as well as more detailed between-emotion analyses of agreement (Harley et al., 2015). When comparing results from self-report and facial expressions, we found a relatively high overall agreement rate of 75.6% when similar self-reported emotions were grouped together along theoretical dimensions and definitions (e.g., anger and frustration). Agreement varied considerably, however, depending on the emotion in question. Our range of agreement included 84% for happiness and 7.14% for surprise, highlighting the emotion-dependent nature of agreement between self-report and facial expression recognition software. Our results concerning agreement of emotional states when using skin conductance were lower, with overall agreement rates of 60% (facial expressions) and 41% (self-report), though variation was observed between emotional states and self-reported endorsement levels. Of particular note, agreement levels were more than 10% higher when using Likert response items rated at the high end of the scale (5) compared to 4 or the midpoint (3). This study contributed to a small corpus of research examining coherence in emotional expressions and provided novel methodological approaches to aligning and comparing emotions in long experimental sessions, in contrast to shorter experimental trials that were more typical (Mauss et al., 2005).

#### Did Learners’ Traits Influence How They Felt? and Did These Feelings Differ by Object Foci?

We have also examined the role of key individual differences in predicting learners’ emotions, and not just general emotions: those elicited from attending to different MetaTutor object foci, the four PAs. Significant relationships between a subset of trait emotions (trait anger, trait anxiety) and personality traits (agreeableness, conscientiousness, and neuroticism) were found for four agent-directed emotions (enjoyment, pride, boredom, and neutral), though the relationships differed between virtual PAs (Harley et al., 2016a). These results, along with those from a follow-up study examining goal orientations (Lallé et al., 2017a,b, 2018, 2021) were critical in establishing the need to contextualize the source of emotion in considering emotional interventions and the design of virtual PAs.

### Lessons Learned and Future Directions

These studies highlight a number of limitations and directions for future research. Theoretically, the CVT (Pekrun, 2006; Pekrun et al., 2011) provides valuable insight regarding sources of and processes involved in emotion generation but does not provide a detailed account of how emotions can be regulated. If research is to leverage the benefits and minimize the negative impact of emotions on academic achievement, additional theoretical guidance is needed. Fortunately, such a theory has recently been developed that integrates and extends propositions from the CVT and process model of emotion regulation (Gross, 2015): the emotion regulation in achievement situations (ERAS; Harley et al., 2019b). Though describing this theory in detail is beyond the scope of this article, ERAS provides propositions and examples about the differential effectiveness of five families of emotion regulation strategies when (a) they are implemented across achievement situations with different characteristics (individual vs. social and high- vs. low evaluative axes), (b) situations are contextualized by different object foci and time frame perspectives, and (c) different discrete emotions are targeted for regulation. In doing so, the ERAS model stands to help reveal the complexities and nuances of how emotions are regulated in achievement situations and shine a light on key affordances and constraints associated with their regulation in emerging literature.

Methodologically and analytically, these studies highlight that more research is needed for assessing different object foci, especially for complex intelligent technologies like MetaTutor, in order to better understand the relative contributions of different aspects of an environment to the emotions learners experience. We examined how learners with different personality and emotional dispositions felt about each of the four PAs, but what about the SRL palette? The educational content of different multimedia, etc.? Results from a separate program of research on emotions experienced with mobile apps provide supporting evidence that emotions and appraisal mechanisms can differ between discrete aspects of technology-rich learning environments (Harley et al., 2016a,b, 2019a,2019b,^2020^). Future research should therefore extend emotion analyses from general retrospective accounts and even moment-specific concurrent self-reports to specific object foci. This can be accomplished through self-report measures, such as the multiple object foci emotion questionnaire (MOFEQ; Harley et al., 2016a,b, 2019a,2019b,^2020^), that ask about specific aspects of an environment or inferred from using eye-tracking data that capture where someone was looking when an emotion was experienced.

Our results also highlighted a substantial amount of neutral affect and a limited range of emotional states (e.g., low levels of frustration). Low levels of intensity stand to make detecting emotions through facial expression recognition software and physiological measurements more challenging. Thus, a future direction for research on alignment may be to endeavor to align learning session content with learners’ academic degrees to enhance appraisals of value. Another promising direction for future research is to integrate emotion regulation prompts into intelligent systems like MetaTutor, perhaps using a fifth agent, Elly the emotion regulator.

## Motivation During Learning With MetaTutor

What drives the effort invested into a task? How might different achievement goals impact learners’ approach and response to the MetaTutor environment? Such questions relate to the motivational facets of SRL (Zimmerman and Schunk, 2011; Usher and Schunk, 2018; Renninger and Hidi, 2019). Motivation has been studied within MetaTutor primarily by assessing learners’ achievement goals for the learning task. In line with Achievement Goal Theory (Elliot and Murayama, 2008), learners complete a brief questionnaire to assess the extent to which they adopt the following orientations: mastery-approach, performance-approach, mastery-avoidance, and performance-avoidance. Goal orientation refers to a learner’s purpose or aim for an achievement task. The goal may be to improve knowledge (mastery orientation), to perform better than others (performance-approach orientation) or to avoid failure relative to others (performance-avoidance orientation). While a combination of mastery and performance goals may be ideal for learning and achievement, mastery goal orientation is typically associated with desirable outcomes, such as high engagement, intrinsic motivation, and persistence (Linnenbrink-Garcia et al., 2016), whereas performance approach orientation has been more consistently linked with achievement (Hulleman et al., 2010b).

Research conducted to date on motivation within MetaTutor has examined how different achievement goal orientations interact with PA supports to impact SRL processes and learning outcomes (Duffy and Azevedo, 2015). For instance, if learners are more motivated to improve their knowledge, do they approach the task differently than learners driven primarily by a desire to outperform peers? Do learners with different motivations react in distinct ways to prompts and feedback? Given that achievement goals provide an overarching aim for learning tasks that direct and guide behaviors, they are expected to impact SRL processes, as well as subsequent learning outcomes (Pintrich, 2004). For instance, mastery-oriented learners may be driven by their own curiosity or desire to enhance their understanding of a topic, which may lead them to focus on material deemed most interesting at the expense of other content (Senko and Miles, 2008). On the other hand, performance-oriented learners may be more concerned with how they perform relative to others and therefore focused on covering all to-be-tested material, complying with prompts and feedback that help them to realize this goal.

While we have utilized Winne and Hadwin’s (1998) theory of SRL extensively to guide our research in cognitive and metacognitive processes, here we elaborate briefly as to how this same theory has been used as a guiding framework for research on achievement goals and SRL in MetaTutor. Within this framework, achievement goals are most recognizable within the first two phases of learning: (1) *task definition;* and (2) *planning and goal setting*. For example, a mastery goal learner may perceive the task to be an opportunity to improve understanding and depth of knowledge about the circulatory system (task definition), which in turn may lead them to set a goal to improve their knowledge about a specific sub-topic of interest and create a plan to focus on this material (planning and goal setting). In contrast, a performance goal learner may perceive the task to be an opportunity to outperform peers (task definition), leading them to set a goal to attain the highest score on the test and a plan to cover as much testable material as efficiently as possible (planning and goal setting). Similarly, social cognitive models of SRL (Pintrich, 2000, 2004; Zimmerman, 2011) identify motivational variables, such as achievement goals, within the *forethought* phase. In our research on the role of motivation in MetaTutor (Duffy and Azevedo, 2015), we also posited that achievement goals activated during these initial SRL phases are likely to influence subsequent *enactment* and *adaptation* stages of SRL by influencing learner perceptions and responsiveness to PA prompts and feedback. In other words, an interaction is likely to occur between motivational profiles and PA scaffolds within MetaTutor.

### Synthesis of Findings

The findings from MetaTutor studies have largely shown that motivation indeed plays a role in learning processes and outcomes. For instance, Duffy and Azevedo’s (2015) study demonstrated a significant interaction effect between PA condition (prompt and feedback vs. control) and achievement goal (performance-approach vs. mastery approach), such that learners with a performance-approach goal significantly outperformed learners with a mastery-approach goal on the post-test, but only in the condition in which learners received PA scaffolding for SRL (prompt and feedback condition). In the condition without PA support for SRL (control condition), motivational profiles had no impact on learning outcomes, which suggests that learners with different achievement goals react differently to PA scaffolding. This finding is consistent with a growing body of research that has found mastery-approach goals less consistently linked to performance compared to performance-approach goals (Senko et al., 2011). Why did performance-approach learners benefit from the scaffolds but mastery-approach learners did not? One explanation is that mastery-approach learners set self-referential goals (self-improvement), which may lead to less demanding conditions for success than those with a performance-approach who aim to obtain the highest score on the test compared to others. Linking back to Winne’s model (Winne’s, 2018), this suggests that the achievement goal influences the *standards* for success in SRL. Additionally, mastery learners may perceive PA scaffolds to be misaligned with their learning agenda and more of a distraction or interference in their goal pursuit, whereas performance learners may perceive agent scaffolds as helpful in realizing their goals.

Accordingly, we hypothesized that learners with a mastery-approach goal may have had more negative reactions to PAs’ prompts and feedback. Exploratory case analysis of two mastery-approach learners (one from the prompt and feedback and one from the control condition) was conducted using think-aloud and facial expression data to explore whether differences emerge in response to PA scaffolds (Duffy and Azevedo, 2013). Preliminary analysis revealed the mastery-approach learner in the prompt and feedback condition demonstrated more negative emotions, whereas the mastery-approach learning in the control condition experienced more positive emotions. Subsequent MetaTutor studies sought to test this hypothesis directly (e.g., Lallé et al., 2016; Lallé et al., 2017a,b) and reported consistent findings. Specifically, Lallé et al. (2016) results revealed that performance-approach learners reported more pride and less anxiety in the prompt and feedback condition than in the control condition, whereas mastery-approach learners reported the opposite pattern: more anxiety and less pride in the prompt and feedback condition than in the control condition. Further, evidence from eye-tracking data (Lallé et al., 2017b) demonstrated that performance-approach learners showed improved learning outcomes when fixating *longer* and at a higher rate on PAs (i.e., attended more to PAs), whereas mastery-approach learners again showed the opposite pattern: they benefited when attending *less* to PAs.

Another study by Cloude et al. (2021a,b) investigated the degree to which learners engaged in metacognitive judgments initiated on pages containing information relevant to achieving either sub-goals 1 or 2. Specifically, 186 undergraduates’ multimodal data were captured during learning and analyzed using latent growth models. Results showed that the stability (such that it did not increase) of page-irrelevant metacognitive judgments from the first to second sub-goal was positively related to performance, but there were no relations between achievement goal orientation and these variables. Additionally, there were no relations between page-relevant metacognitive judgments across sub-goals 1 and 2, achievement goal orientation, and performance. This study provides another example of examining motivation in relation to process data. Future research utilizing this method could provide insight into designing effective interventions based on what personally motivates learners to engage in metacognition to augment their learning and performance with emerging technologies (Cloude et al., 2021a,b).

### Lessons Learned and Future Directions

Taken together, these findings suggest that learners with different motivational profiles are likely to perceive and react to prompts and feedback differently, which in turn bears on instructional design. Whereas performance-approach learners benefited from PA support, mastery-approach learners did not. Self-determination Theory (SDT) can help to explain these distinct patterns and in particular why some learners may have less positive reactions to PAs. According to SDT, when an individual’s basic psychological needs are thwarted (e.g., feels that their need for competence or autonomy is impeded), the individual is likely to react negatively to regulation efforts, whereas when these needs are met, they are likely to react positively, given that the regulation is more internalized (e.g., Ryan and Deci, 2000; Niemec and Ryan, 2009; Deci and Ryan, 2011). It may be the case that performance-approach learners find the PAs supportive of their needs, whereas mastery-approach learners perceive them to be more controlling. This is consistent with research that has found mastery-approach goals to be linked with more positive emotions and engagement in autonomy-supportive environments (Benita et al., 2014).

The findings on motivation in MetaTutor have several implications for the design of advanced learning technologies. Although the current features appear to be adaptive for performance-approach learners, those with other motivations could also be supported. First, it would be useful to modify intelligent tutor systems so that they are adaptive to a more diverse array of motivational profiles. To benefit mastery-approach learners, agents may need to provide different types of prompts and feedback that take into consideration their values while also communicating the importance of the scaffolds in achieving their goals. This may help these learners to view PA scaffolds for SRL as a support rather than a distraction. This is consistent with Expectancy-value Theory of Motivation (Wigfield and Eccles, 2000) and corresponding value-based interventions (e.g., Hulleman et al., 2010a), which suggest that increasing utility appraisals (and reducing cost appraisals) will enhance achievement. Second, PA scaffolding could be designed to enhance users’ sense of autonomy by allowing learners to select the frequency and type of feedback delivered, in line with an open learner model (Bull, in press). Mastery-approach learners, in particular, may also benefit from less frequent agent interaction or from fading scaffolds over time (Belland, 2013). A key feature of the MetaTutor studies described is that they illustrate how self-report data of motivation can be examined alongside trace data to provide a richer understanding of both the motivating *goals* (questionnaires) and resultant learning *processes* (eye-tracking, log files). This was examined further in Cloude et al. (2021b) by investigating trace data and its relation to different sub-goals over the course of the learning session, which proved useful in identifying signatures of goal pursuit, potentially providing future direction for implicit measures of motivation in action rather than as a one-point-in-time assessment, which we discuss next.

From a theoretical perspective, there are several motivational frameworks that have been used to guide interpretation of findings but not yet directly tested in MetaTutor, including EVT and SDT. As previously noted, these theories could help to inform design changes in the delivery of agent scaffolding to enhance the perceived utility and internalization of SRL prompts. Examining learners’ satisfaction and attributions for learning outcomes could also help us to better understand how motivation influences the standards of SRL. In terms of methodological advancements, it would be helpful to include unobtrusive measures of motivation at a finer-grained unit of analysis to examine stability and change over time. Existing MetaTutor studies involving motivation have focused on examining learners’ self-reported motivation in relation to traces of learning processes. However, MetaTutor captures other types of trace data (e.g., time on task via log files) that can serve as a proxy to track other facets of motivation not addressed here, such as effort, persistence, and choice, especially to study its relation to different sub-goals over time during the learning session. This could provide more information about the *degree* of motivation, whereas data analyzed to date has focused on the *type* of motivation. Additionally, think-aloud data could also be examined for indicators of curiosity, interest, and self-efficacy. Together, these traces could provide insight into the dynamic nature of motivation. Finally, limited research has examined the regulation of motivation (Wolters, 2003; Schwinger and Stiensmeier-Pelster, 2012), which includes understanding how learners monitor and deploy strategies to boost or sustain motivation. To fully understand the role of motivation in learning, a key step will require examining motivation as the target of regulation, and non-linear dynamical systems (NLDS) could offer the tools to do just that (Gabriel et al., in press). NLDS explains dynamics that occur within a system of interconnected elements like SRL that undergoes change (Schuster, 1984; Guastello et al., 2008). In the next section, we describe the recent extension MetaTutor in addressing college students’ learning disabilities and contributions of our research on MetaTutor.

## Current Extensions of MetaTutor—MetaTutorEs

Recently, Dr. Cerezo and her team in Spain have created a Spanish version of MetaTutor, MetaTutorES, and conducted several studies with college students with learning disabilities, including the development of a multimodal evaluation protocol for adults with learning disabilities based on MetaTutor (Cerezo et al., 2020b). Their recent study (Cerezo et al., 2020a) examined how 119 college students both with and without learning disabilities regulate their learning with MetaTutorES. Results showed that those in the experimental group (i.e., provided with adaptive scaffolding from the PAs) used more system-initiated and self-initiated self-regulation strategies than those in the control group. In addition, all students showed some improvement in learning from pre to posttest. The results showed that students with learning disabilities can take advantage and benefit from embedded tools such as PAs’ prompting and scaffolding to learn complex science topics.

In a subsequent study, Cerezo’s team (Chango et al., 2021) collected and preprocessed data from 40 students using different multimodal sources: learning strategies from log files, emotions from videos of facial expressions, allocation and fixations of attention from eye tracking, and performance on posttests of domain knowledge. They used multimodal data to test whether the prediction could be improved by using attribute selection and classification ensembles of the students’ processes. They carried out three experiments by applying six classification algorithms to numerical and discretized preprocessed multimodal data. The results showed that the best predictions were produced using ensembles and selecting the best attributes approach with numerical data. These findings have implications for early detection of students’ challenges in self-regulating their learning using multimodal data.

## Contributions to the Field of Self-Regulated Learning and Intelligent Learning Technologies

Our extensive research on MetaTutor has contributed to current theoretical models of SRL, methodological approaches to studying SRL, and analyses of SRL processes underlying self-regulation during complex learning (Tarricone, 2011; Veenman, 2011; Greene et al., 2015; Järvelä and Bannert, 2019; Winne, 2019; Azevedo, 2020; Lajoie et al., in press). Despite these contributions, there are several theoretical, conceptual, methodological, and analytical limitations that need to be addressed in future research. For example, can we develop a comprehensive model or framework of SRL that integrates CAMM processes in a way that contributes to our understanding of each process and their combined role in self-regulation over time? What do behavioral traces of qualitative changes in metacognitive monitoring look like? Do they reside in specific trace data (e.g., concurrent verbalizations are needed to understand metacognitive monitoring along with log files to measure the duration of learning strategies) or are they evident across multiple data channels (e.g., physiology + facial expressions + screen recordings are needed to understand emotion regulation strategy use)? Are dynamical systems approaches (e.g., growth modeling, recurrence quantification analysis, etc.) better suited for analyzing the temporal and complex nature of SRL processes using multimodal data (see Favela, 2020)? If so, how can they better theoretically explain the temporal dynamics of CAMM processes and can such analyses be used to better design multi-agent intelligent systems capable of triggering more accurate pedagogical interventions through PAs? Below we pose specific theoretical, conceptual, methodological, and analytical questions that should drive future research.

Theoretically, there are key CAMM-specific questions that need to be addressed. For example, Winne’s (2018) model mentions that cognitive and task conditions impact students’ use of metacognitive processes, however, it does not pinpoint how or when specific metacognitive processes are used during learning. As another example, the model of metamemory (Nelson and Narens, 1990) does emphasize the use of specific metacognitive judgments (e.g., EOLs, JOLs, retrospective confidence judgment). However, this can limit analyses to only examining some metacognitive judgments when it is possible students are engaging in other metacognitive processes (e.g., Greene and Azevedo, 2009; Azevedo and Dever, in press). The same argument can be made for motivation and affective states when it comes to describing the key constructs, processes, and mechanisms for CAMM processes. Can we develop and test a unified model of CAMM SRL that is complete, affords predictions, and allows researchers to generate research questions and testable hypotheses across learners, tasks, domains, and contexts? The underlying assumptions of such a comprehensive model could be embodied in systems like MetaTutor. For example, an interface designed to facilitate cognitive processing of multiple representations of information (Azevedo and Taub, 2020) and where the STEM content can dynamically change to account for fluctuations in motivational states by providing additional diagrams due to sustained interest in the topics detected from verbalizations, physiological sensors, and prolonged fixations. A system that includes intelligent PAs capable of integrating facial expressions with natural language processing (NLP) to detect students’ emotion regulation strategy-use and providing adaptive emotional regulation scaffolding, when necessary, which may include modeling emotion regulation strategies. Also, the system could include negotiable open learner models triggering metacognitive awareness and affording students opportunities to calibrate their own metacognition by overriding the system’s beliefs of their metacognitive skills. Any of the system’s features can be experimentally manipulated to show the impact of CAMMs on SRL in advanced learning technologies such as MetaTutor.

Conceptually, results from our studies do not have a common consensus regarding the ideal time to engage in CAMM processes. For example, when it comes to metacognition it can be argued that in MetaTutor, a student should first assess the relevance of the page, and if relevant, judge their understanding of the text before engaging in cognitive learning strategies. However, this would take a large amount of cognitive effort, leaving little time to actually inspect and learn the material. The same argument can be leveled at cognitive, affective, and motivational processes. For example, what should a system do if it detects that students are experiencing issues with all CAMM processes? Which process does the system prioritize? Do we address the motivational and affective issues first and then proceed to the cognitive and metacognitive processes? Or does the system tackle all of them together and if so, what does it look like and what is the theoretical basis for such decisions? Thus, it remains unclear the ideal amount and sequence of engaging, or scaffolding engaging, in these processes.

Another conceptual issue relates to providing different types of scaffolding and support to students who are under- vs. over-confident, or inaccurate at making metacognitive judgments (Azevedo and Wiedbusch, in press). For example, if a student is over-confident, they will require different types of support compared to a student who is under-confident. A student who performs poorly on a quiz, but judged greater understanding is inaccurate and over-confident, and support would need to focus on helping the student acquire the domain knowledge for that content, in addition to knowledge on how to metacognitively judge their understanding. Conversely, a student who performs well on a quiz, but judged less understanding is under-confident, so the support should perhaps focus on procedural or conditional knowledge because they have demonstrated they have the domain knowledge. As a third example, a student who has a low performance, but accurately judged this will only need support for acquiring the domain knowledge. Thus, it is important to understand a student’s domain knowledge in addition to their procedural or conditional metacognitive knowledge to ensure they are acquiring both sets of skills. Perhaps MetaTutor’s production rules will be able to account for these different levels of knowledge in future iterations. Again, similar questions can be posed about emotions and motivation, which MetaTutor is currently not capable of scaffolding but can clearly be modified to address. For example, how do we scaffold task value, interest, self-efficacy, cognitive reappraisals, should a new agent (e.g., Megan the motivator) be created to support the regulation of emotion and motivation etc.?

Methodologically, using log files and eye-tracking to examine metacognitive processes is advantageous because they are unobtrusive and unbiased measures. However, this also requires us to make inferences that students are, in fact, engaging in metacognitive processes. For example, when students select a JOL or FOK from the SRL palette, are they really judging their understanding or familiarity with the text, or are they self-testing (i.e., want to take the 3-item quiz)? Also, through the SRL palette, we only measure processes that are externalized either verbally or behaviorally or both by the student. For instance, a JOL selected from the palette on one page might be followed by further “internal” (i.e., not uttered or behaviorally enacted) JOLs on subsequent pages, that we can’t measure without triggering, prompting, or interfering with the process. Additionally, when we used eye-tracking data as indicators of monitoring behaviors (e.g., AOI-pair from text to the timer or sub-goal progress bar indicating monitoring progress), how can we be sure students are monitoring their behaviors, as opposed to looking around because they are bored or frustrated? This demonstrates the need for using multimodal multichannel data to investigate all CAMM processes together, and how different channels can be used as indicators of each process (Azevedo et al., 2019; Azevedo and Wiedbusch, in press). Additional methodological issues to be addressed in the future include identifying the right suite of tools, devices, and sensors, required to measure CAMM processes in laboratory experiments, with particular constraints if we want to ensure that this suite can be portable, scalable, etc. to be applied to other non-lab contexts (e.g., classrooms, immersive virtual learning, informal settings). How does adapting the suite of tools to the different non-lab contexts impact the quality of research, data, and what analytical challenges does it create and what are the implications for the development of a comprehensive unified theory of SRL (Biswas et al., 2018)?

Analytically, our research has made great progress in moving toward using educational data mining techniques, such as cluster analysis and sequence mining (Bouchet et al., 2012, 2013; Taub et al., 2014; Taub and Azevedo, 2019) to examine metacognitive and cognitive behaviors during learning with MetaTutor as opposed to relying exclusively on traditional inferential statistics that combine event data into a single event per participant. We have also used unsupervised machine learning techniques to examine (Lallé et al., 2018, 2021; Wortha et al., 2019; Wiedbusch and Azevedo, 2020) complex eye-tracking data and facial expressions of emotions during learning with MetaTutor. We continue to use non-traditional statistical techniques, including dynamical systems modeling (Dever et al., in press) to examine learners’ emergent SRL behaviors, and MLAs to predict performance at the end of the learning session (Mu et al., 2020; Saint et al., 2020; Chango et al., 2021; Fan et al., 2021). Despite our ability to continuously adapt and use contemporary analytical techniques that emerge from the computational, engineering, psychological, statistical, and data sciences, we as a field are still faced with a major barrier that continues to impact the educational effectiveness of intelligent systems such as MetaTutor. The issue is that these analyses are all conducted in a *post-hoc* fashion (i.e., *after* a student learns with MetaTutor), thus moving forward, it would be beneficial to analyze these processes in real-time, and provide truly intelligent, adaptive personalized support of CAMMs. Machine learning approaches are particularly promising in this regard as their focus lies in the prediction of behavior rather than (post hoc) explanations (Yarkoni and Westfall, 2017). Further, they generally are capable of addressing issues of traditional statistical analyses with regards to adequately handling large amounts of data (e.g., Dwyer et al., 2018), such as the multichannel data collected with MetaTutor. Thus, machine learning models, trained on multi-channel data, can serve as the basis for increasingly adaptive systems that can intervene in the learning processes as or before issues arise. In addition, modeling approaches that bridge the gap between theory driven psychological analyses and data driven machine learning approaches would be very beneficial for future adaptive systems. In sum, these are some of the major issues that need to be addressed by future research (see also Azevedo and Gašević, 2019; Järvelä and Bannert, 2019; Winne, 2019; Azevedo, 2020; Lajoie et al., 2020; Hadwin, 2021; Li and Lajoie, in press).

## Lessons Learned and Open Challenges

In sum, there are some lessons learned and open challenges for interdisciplinary researchers. First, SRL takes time to develop and needs to be acquired, internalized, and practiced over time with the assistance of human and artificial agents to enhance learning and transfer. Therefore, future intelligent systems may need to scaffold learning and should encourage students to interact with such systems for a longer period of time. Second, adaptive (intelligent) scaffolding is key to supporting students’ SRL with learning technologies, but this can only be achieved once we understand how CAMM processes dynamically and temporally unfold and how they relate, contribute, and impact real-time learning processes (Hadwin, 2021). To do so, it is critical that system features become more seamless in their interactions with students (e.g., hold a conversation using NLP) and use stealthier assessment (gaze-behavior analysis, etc.) to adapt itself to the needs of each individual student. If theory suggests and assumes that learning is a dynamic process that is cyclical and non-linear, the methods in which we capture and measure learning should reflect this as well as the design of future system architectures. Third, multimodal multichannel SRL CAMM data is key to understanding the dynamics of SRL during learning, problem-solving, reasoning, understanding, etc. Additional tools, methods, sensors, and techniques may be needed in the future to increase the accuracy, reliability, and validity of detecting and measuring these processes and validate the inferences researchers make about these processes to hopefully reduce the inference/increase accuracy coefficient so that intelligent systems like MetaTutor provide optimal just-in-time scaffolding. Fourth, we argue that the concepts of meta-learning, meta-thinking, and meta-reasoning from the psychological and computational sciences are key to acquiring, internalizing, using, and transferring SRL knowledge and skills across tasks, domains, and contexts (Cox, 2011; Cox et al., 2016; Ackerman and Thompson, 2017). Fifth, data visualizations of students’ multimodal SRL processes are key to enhancing their understanding of SRL, just as visualizations are key in designing teacher dashboards that provide actionable data for effective instructional decision-making thus creating a human-AI complementarity (Molenaar and Knoop-van Campen, 2019; Holstein and Aleven, 2021; Wiedbusch et al., 2021b). Sixth, while we acknowledge that cognition, metacognition, and emotions are important for SRL, more attention needs to be paid to the role of motivation (as states that also fluctuate during task performance, perhaps at different time epochs, and that can be deeply intertwined to the concept of flow (Csikszentmihalyi et al., 2018). Seventh, training teachers to learn and use SRL in their classrooms is key in fostering their students’ SRL (Kramarski, 2018; Callan and Shim, 2019; Dignath and Veenman, 2020; Kramarski and Heaysman, 2021) and must remain a major thrust of research and education in our field. Lastly, AI-based immersive virtual environments hold great promise to enhance students’ SRL, especially with the use of AI, NLP, computer vision, and machine learning and nanomaterials (e.g., sensors) that can significantly advance and address conceptual, theoretical, methodological, analytical issues and have a major education impact on students of all ages.

## Author Contributions

RA significantly contributed to the conceptualization, construction, and writing of the entire manuscript, especially the last three sections of the manuscript, integrated, synthesized, and finalized the manuscript. FB conceptualized, contributed, and wrote the MetaTutor architecture section. MD conceptualized and synthesized the studies presented in the motivation section. JH conceptualized and synthesized the studies presented in the emotion section. MT conceptualized and synthesized the studies presented in the metacognition section. GT conceptualized and synthesized the studies presented in the cognitive section. EC, DD, MW, and FW contributed to all the sections of the manuscript by synthesizing recent work on MetaTutor. RC contributed to the section of MetaTutorES. All authors were involved in the review and editing of the final version of the manuscript.

## Author Disclaimer

Any opinions, findings, conclusions, or recommendations expressed in this material are those of the author(s) and do not necessarily reflect the views of the National Science Foundation.

## Conflict of Interest

EC was employed by the Soar Technology, Inc. The remaining authors declare that the research was conducted in the absence of any commercial or financial relationships that could be construed as a potential conflict of interest.

## Publisher’s Note

All claims expressed in this article are solely those of the authors and do not necessarily represent those of their affiliated organizations, or those of the publisher, the editors and the reviewers. Any product that may be evaluated in this article, or claim that may be made by its manufacturer, is not guaranteed or endorsed by the publisher.
